# 468. Assessment of The Proportion of Hospitalized Patients (Pts) with Candidemia (C) and Invasive Candidiasis without Candidemia (IC) Who Received an Echinocandin (EC) and Were Potentially Eligible for an Earlier Hospital Discharge (HD)

**DOI:** 10.1093/ofid/ofac492.526

**Published:** 2022-12-15

**Authors:** Thomas Lodise, Kevin W Garey, Brian H Nathanson

**Affiliations:** Albany College of Pharmacy and Health Sciences, Albany, New York; University of Houston, Houston, TX; OptiStatim LLC, Longmeadow, Massachusetts

## Abstract

**Background:**

The primary driver of costs for C/IC pts is hospital length of stay. Studies across multiple infections demonstrate that most clinically stable pts with modest diagnostic & therapeutic requirements can be safely discharged prior to actual HD day. Few studies have assessed if there is an opportunity to accelerate time to HD in pts with C/IC. This study sought to determine the proportion of US hospitalized adult pts with C/IC who received an EC near HD & was potentially eligible for an earlier HD.

**Methods:**

Design: Retrospective, multi-centered observational study using Premier Healthcare Database (1/2016-4/2019). Study criteria: hospitalized; age ≥ 18 years; *Candida sp*. on a culture consistent with C/IC; ≥3 days of an EC for C/IC; discharged alive; & received an EC near HD (-2 day to HD day). Pts were considerable potentially dischargeable if they met the following 3 criteria & maintained these 3 criteria until HD: resided on a non-ICU hospital ward, taking oral medications, & had no receipt of any diagnostic/therapeutic interventions (insertion of PICC lines were permitted). The difference in hospital days between first potentially eligible HD day & actual HD day was quantified. The proportion of pts that was potentially eligible for an earlier HD was examined overall & by Charlson Comorbidity index (CCI), C/IC, & *Candida sp*.

**Results:**

During study period, 1,599 pts received an EC ≥ 3 days for C/IC & were discharged alive. Of the 1,599 pts, 1,008 (63%) were on an EC near HD. For the 1,008 pts on an EC near HD, the mean (SD) age was 59 (16) years, 52% were male, 40% had a CCI ≥4, 35% were in the ICU on index C/IC culture day, & 64% had C vs IC. *C. glabrata* (31%) & *C. albicans* (31%) were the most frequent *Candida. sp*. Of the 1,008 pts on an EC near HD, 14%, 21%, 29%, & 38% were potentially dischargeable 4, 3, 2, & 1 day(s), respectively, prior to the actual HD day (**Figure**). The proportion of pts who were potentially eligible for HD at least 2 days prior to actual HD day did not vary by CCI score, C/IC, & *Candida sp*.

**Figure:**
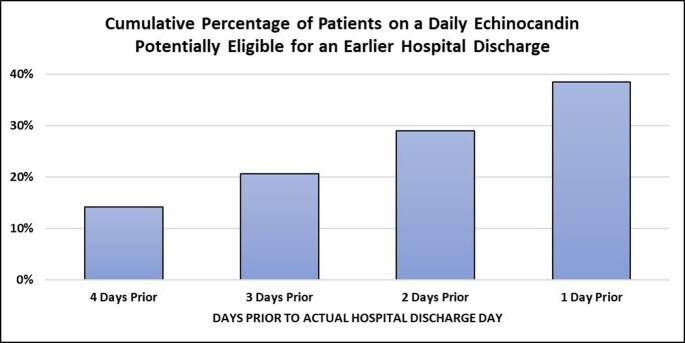

**Conclusion:**

Our findings suggest that a high proportion of hospitalized pts with C/IC receiving an EC near the time of HD, had modest diagnostic/therapeutic requirements prior to actual HD day & were potentially eligible for an earlier HD regardless of CCI, infection type, or *Candida sp*.

**Disclosures:**

**Thomas Lodise, Jr., Pharm.D., PhD**, BioFire Diagnostics: Grant/Research Support|cidara: Advisor/Consultant|cidara: Honoraria|Entasis: Grant/Research Support|Merck: Advisor/Consultant|Merck: Grant/Research Support|Paratek: Advisor/Consultant|Shionogi: Advisor/Consultant|Spero: Advisor/Consultant|Venatrox: Advisor/Consultant **Kevin W. Garey, PharmD, MS**, Acurx: Grant/Research Support|cidara: Advisor/Consultant|cidara: Grant/Research Support|Paratek: Grant/Research Support|Seres Health: Grant/Research Support|Summit: Grant/Research Support **Brian H. Nathanson, Ph.D.**, cidara: Grant/Research Support|Merck: Advisor/Consultant|Merck: Grant/Research Support.

